# “Facilitating HIV status adjustment: Qualitative insights from the Tambua Mapema proof-of-concept study in Kenya”

**DOI:** 10.1371/journal.pone.0261255

**Published:** 2022-01-13

**Authors:** Elise M. van der Elst, Mitchelle Abuna, Clara Agutu, Fred Ogada, Aisha Galole, Joyce Shikuku, Tony Oduor, Susan M. Graham, Eduard J. Sanders, Don Operario

**Affiliations:** 1 Department of Clinical Sciences, Kenya Medical Research Institute—Wellcome Trust Research Programme, Kilifi, Kenya; 2 School of Public Health, Brown University, Providence, Rhode Island, United States of America; 3 Departments of Medicine, Global Health, and Epidemiology, University of Washington, Seattle, WA, United States of America; 4 Centre for Tropical Medicine and Global Health, University of Oxford, Headington, United Kingdom; Udayana University: Universitas Udayana, INDONESIA

## Abstract

Systematic efforts are needed to prepare persons newly diagnosed with acute or chronic HIV infection to cope. We examined how patients dealt with this news, looking at how readiness to accept an HIV diagnosis impacted treatment outcomes, prevention of transmission, and HIV status disclosure. We examined vulnerability and agency over time and considered implications for policy and practice. A qualitative sub-study was embedded in the Tambua Mapema (“Discover Early”) Plus (TMP) study (NCT03508908), conducted in coastal Kenya between 2017 and 2020, which was a stepped wedge trial to evaluate an opt-out HIV-1 nucleic acid testing intervention diagnosing acute and chronic HIV infections. Diagnosed participants were offered antiretroviral therapy (ART), viral load monitoring, HIV partner notification services, and provision of pre-exposure prophylaxis (PrEP) to their uninfected partners. Data were analyzed using thematic approaches. Participants included 24 individuals who completed interviews at four time points (2 weeks and 3, 6, and 9 months after diagnosis), including 18 patients (11 women and 7 men) and 6 partners (1 woman, 5 men, of whom 4 men started PrEP). Acceptance of HIV status was often a long, individualized, and complex process, whereby participants’ coping strategies affected day-to-day issues and health over time. Relationship status strongly impacted coping. In some instances, couples supported each other, but in others, couples separated. Four main themes impacted participants’ sense of agency: acceptance of diagnosis and commitment to ART; positive feedback after attaining viral load suppression; recognition of partner supportive role and focus on sustained healthcare support whereby religious meaning was often key to successful transition. To support patients with acute or newly diagnosed chronic HIV, healthcare and social systems must be more responsive to the needs of the individual, while also improving quality of care, strengthening continuity of care across facilities, and promoting community support.

## Introduction

Kenya has the fifth largest number of people living with HIV globally. An estimated 1.3 million Kenyans are chronically infected, with 36,000 new adult infections occurring in 2018, and approximately 79.5% of people living with HIV knowing their status in 2018 [1]. Provider-initiated HIV testing and counselling presents some unique opportunities to identify undiagnosed people living with HIV [2], offer HIV treatment with viral load (VL) monitoring, and provide HIV partner notification services (PNS) to extend prevention or treatment options to partners. Standard provider-initiated HIV testing and counselling using rapid antibody tests, however, does not identify patients with acute HIV infection [2,3]. Acute HIV Infection (AHI) is typically defined as the first weeks after HIV acquisition, during which HIV antibodies are undetectable [4]. While some AHI patients remain asymptomatic, most experience an acute illness approximately 2 weeks following infection, and the majority of these patients seek healthcare [5–9]. The emergence of point-of-care HIV-1 nucleic acid testing allows for identification of people with acute as well as chronic infection [10].

The Tambua Mapema Plus trial (TMP) was a proof-of-concept study conducted from 2017–2020 that used a recently developed algorithm to screen adult patients aged 18–39 years for symptoms compatible with acute HIV infection when they presented for care at one of six participating out-patient facilities in the Kenyan coast [11–13]. Patients with a risk score ≥2 (1 point each for age 18–29 years, fever, fatigue, body pains, diarrhea, and sore throat; 3 points for genital ulcer disease) and no previous HIV diagnosis were offered an opt-out HIV-1 NA testing intervention. Newly diagnosed participants and their partners (when identifiable and consenting) were subsequently engaged for a qualitative sub-study to assess experiences with HIV diagnosis, immediate offer of antiretroviral therapy (ART) with VL monitoring, and PNS with referral to HIV treatment or pre-exposure prophylaxis (PrEP) according to HIV test results.

Previously, we established that newly diagnosed participants with acute and early HIV infection require tailored counseling support and systematic interventions during the first several months following their diagnosis to facilitate HIV status acceptance, improve care outcomes, and facilitate disclosure to partners and other potential sources of support [14]. A preliminary conceptual framework was proposed and included: 1) understanding and accepting one’s status; 2) developing healthy strategies and adjusting to the reality of the new status; and 3) protecting oneself and others through treatment initiation [14]. In the present study, we aimed to assess if this preliminary framework for patients with acute and early HIV infection could be extended to chronically infected participants who were newly diagnosed in the TMP study. We discuss participants’ experiences with ART with VL monitoring and PNS leading to ART or PrEP initiation in relation to this HIV status adjustment, examining participants’ different forms of vulnerability and agency revealed over time and considering implications for policy and practice.

## Methods

### Study procedures

Newly diagnosed participants derived from the intervention phase of the TMP study conducted between 2018–2020 [12]. In this study, we evaluated the yield of a targeted HIV-1 testing intervention offered to adults aged 18–39 years who were seeking healthcare at four public and two private health facilities in coastal Kenya, met our AHI risk score, and agreed to enroll. The HIV-1 testing intervention consisted of point-of-testing HIV-1 nucleic acid testing followed by standard rapid antibody tests to distinguish AHI from prevalent HIV [11]. Secondary trial outcomes included linkage to HIV care, ART initiation among index patients (those who have newly diagnosed with HIV), and the yield of PNS [12].

Index patients were offered enrolment into a care cohort at a nearby research clinic, where they received immediate ART, VL testing at enrolment and month 6, and PNS. Partners of index patients who tested positive for HIV were offered ART, and partners who tested negative were offered PrEP when eligible.

**Fig 1:**
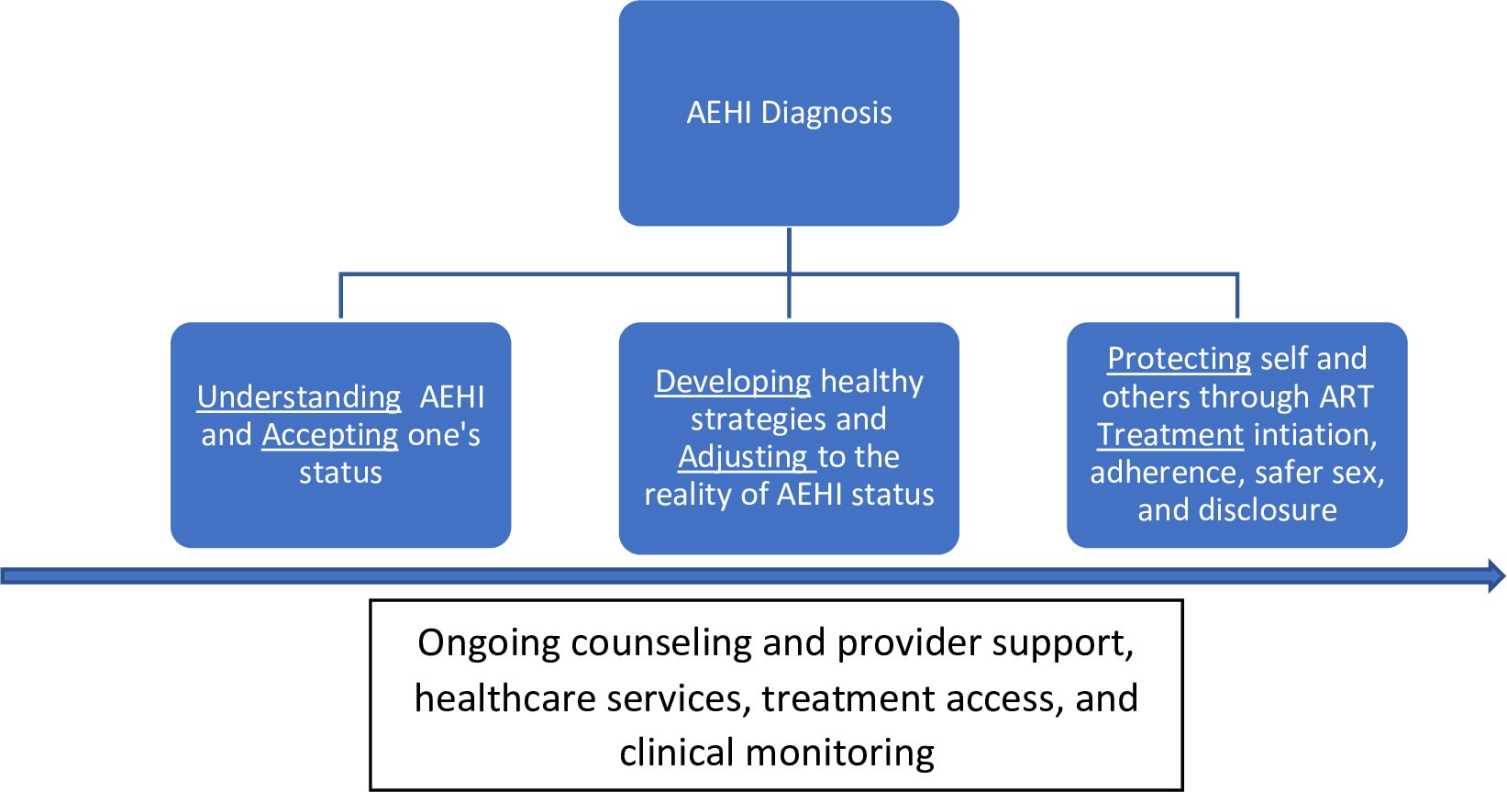
Conceptual model: Adjustment to acute and early HIV (AEHI) diagnosis, ART initiation, and sexual risk reduction (van der Elst et al. 2019) [14].

Qualitative data collection included in-depth interviews (IDIs) with index patients, newly diagnosed HIV-positive partners on ART, and HIV-uninfected partners on PrEP. All IDIs were conducted at the research site in coastal Kenya where HIV services have been offered since 2005. Participants were informed about the study aims, and all participants provided signed informed consent. The IDIs took place at week two, and at months 3, 6, and 9 after diagnosis. Originally, there would have been a closing interview for each patient at month 12, but because of COVID-19 restrictions, the study came to an untimely closure. Interviews covered a broad range of topics informed by theoretical and empirical literature on ART, PrEP, VL testing, and PNS, and incorporated notions from our recently developed conceptual model, corresponding to the acronym U-ADAPT and related to current topics under study, such as ongoing counseling, provider support, healthcare services, treatment access, and clinical monitoring (see Fig 1). VL results -including achieving viral suppression in view of Kenya’s recommendation for HIV-negative partners to be on PrEP for six months- were discussed with both HIV-infected as well as well as HIV-uninfected participants at ART/PrEP start and at month six.

Interview guides were used flexibly to support a rich and relaxed discussion in Kiswahili by native speakers conducting the interviews. To support data quality, interview guides were translated, piloted and revised by the research team prior to data collection. Discussions focused on participants’ experiences, any challenges encountered, coping strategies, and how these strategies were perceived to work. Interview guides were the same for all HIV infected participants. A different IDI was used for the PrEP participants to include specific questions on PrEP experience. The overall aim was to examine participants’ acceptance of (partner’s) HIV diagnosis, looking at treatment uptake and adherence as well as understanding barriers, facilitators, and outcomes of PNS. VL measures at month 6 supported patients’ perspectives on treatment adherence, while patients’ PNS trajectories included HIV disclosure and partnership status.

### Data management and analysis

All interviews were audio-recorded, transcribed verbatim and translated into English. Staff members were instructed to maintain fieldnotes and analytic memos to record observations and elaborate interactions with community members; these were typed up and reviewed for relevant issues to explore in later interviews. Two complementary approaches were applied to analyze the data: the use of a sequential (chronological) matrix [15], and a thematic coding approach [16]. The chronological matrix facilitated exploration of changes over time and patterns of similarity and difference across key themes that emerged. The thematic coding supplemented and enriched the exploration the examination of chronological trends among participants. All transcriptions were coded in NVivo 11 using a coding framework based on our initial and emerging themes of interest, including HIV transition patterns and influences on those patterns. To support the trustworthiness of the coding process, at least two people coded each transcript, comparing results and resolving any discrepancies.

### Ethical considerations

The study received ethical approval by the KEMRI Scientific and Ethical Review Unit (KEMRI/SERU/CGMRC-C/051/3280), the Human Subjects Division at the University of Washington (STUDY00001808), and the Oxford Tropical Research Ethics Committee (OxTREC) at the University of Oxford (Reference: 46–16). The protocol was approved by the Division of AIDS (DAIDS), National Institute of Allergy and Infectious Diseases (NIAID), National Institutes of Health (NIH) (DAIDS-ES 38181). All participants provided written informed consent.

## Results

### Participant characteristics

**Fig 2:**
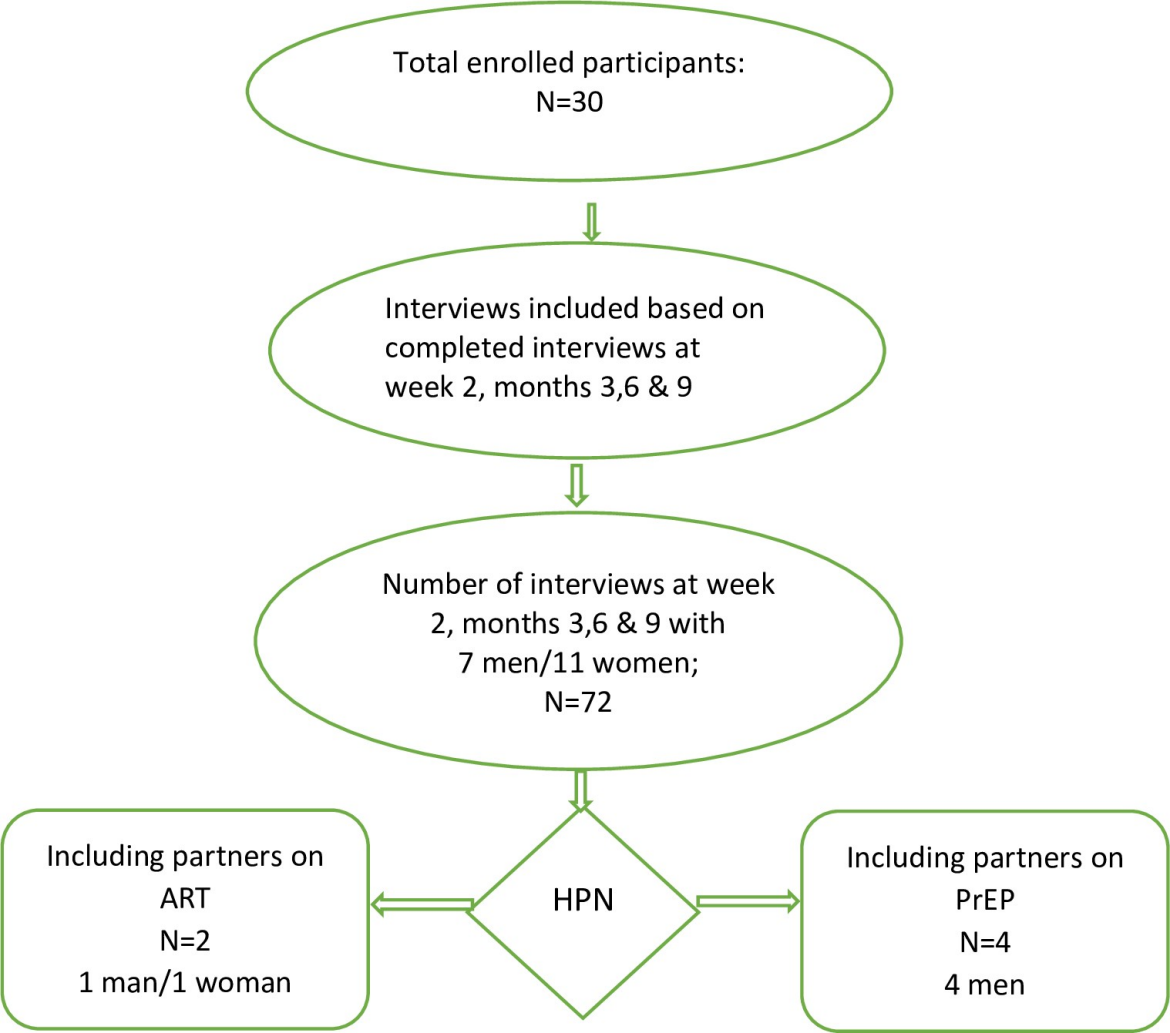
Flow diagram of in-depth interviews with index participants at enrolment, month 3,6, and month 9, including VL measurements at week 6 and month 6; and in-depth interviews with partners.

A total of 30 participants (18 women and 12 men), including two women with acute HIV infection, enrolled at the research clinic [13]. Of those, qualitative interview data from 18 index patients (11 women and 7 men) were analyzed based on completed interviews at week 2 and months 3, 6, and 9 (yielding a total of 72 interviews, Fig 2). Interviewed women had a mean age of 26 years, while the interviewed men had a mean age of 30 years. Approximately half of the participants were or had been married, and 15 participants had primary education or less. Table 1 provides summary characteristics of all sub-study participants. Socio-demographic characteristics of those interviewed did not differ from the index participants not interviewed with respect to age, sex, education and marital status. A total of seven partners were enrolled through PNS, of whom six partners participated in IDIs. These IDIs included two newly diagnosed partners (one man and one woman) who started ART and had their VL measurements taken; and four of the five HIV-negative partners (four men) who initiated PrEP (the woman declined to participate in an IDI). In total, twenty-two partner interviews were obtained, including 14 interviews from PrEP users. One PrEP user discontinued prematurely because the partnership dissolved.

**Table 1:** Socio-demographic characteristics of index HIV-participants; HIV-negative and positive partners, Tambua Mapema Plus trial, coastal Kenya, 2018–2020.

	HIV-index (N = 18)	HIV-negative partners (N = 4)	HIV-positive partners (N = 2)
Sex			
Male	7	4	1
Female	11	0	1
Age (median, IQR)	26 (19,39)	33 (30,33)	35 (26,43)
Education level			
Primary or below	13	1	0
Secondary	3	3	1
Tertiary	2	0	1
Marital status			
Single	5	0	1
Married	11	3	1
Separated/divorced	1	1	0
Widowed	1		
Religion			
Christians	14	3	2
Muslim	4	1	0
None	0	0	0
Source of income			
Employed	5	3	0
Self-employed (casual)	5	1	2
Unemployed			
	8	0	0

### Viral load measures

Table 2 shows VL measures of the index patients at study enrolment and at month 6. At enrolment, the median log_10_ VL for women was 5.0 (range: 0.0–6.9) copies/ml and for men 4.8 (range: 3.2–5.5) copies/ml. At month 6, 16 of 18 index patients were virally suppressed (i.e., <1.6 log_10_, equivalent to <40 copies/ml, or undetectable). One male participant had no change in VL despite self-reported adherence, while one woman reported to have stopped taking ART, which was reflected in an increase of VL from 5.0 to 7.5 log_10_ copies/ml.

**Table 2:** Characteristics of index participants and partners, viral load and partner testing outcomes, Tambua Mapema Plus trial, coastal Kenya, 2018–2020.

No.	Sex	Age	Chronic or acute HIV infection	Log10 Viral load—enrolment	Viral load–Month 6	Regular partners reported	Casual partners reported	Consented to HPN	Positive tests / Number tested	Partner HIV status and linkage outcome
**1**	Female	19	Acute	ND^1^	ND^1^	1	0	Yes	0/1	Spouse (36 years) confirmed negative and initiated on PrEP
**2**	Female	23	Chronic	4.74	<1.60copies/ml	2	1	Yes	0/1	Spouse (33 years) confirmed negative and initiated on PrEP **Couple separated** Regular/casual unknown
**3**	Female	24	Chronic	2.96	Not detected	1	0	Yes	0/1	Spouse (28 years) confirmed negative and initiated on PrEP
**4**	Female	26	Chronic	5.13	7.53 Log10	1	0	Yes		Status unknown
**5**	Female	26	Chronic	5.01	<1.60copies/ml	2	0	Yes	0/1	Spouse status unknown Regular partner (26 years) confirmed negative initiated on PrEP
**6**	Female	26	Chronic	6.06	<1.60copies/ml	1	0	Yes		Spouse died of HIV
**7**	Female	26	Acute	6.91	<1.60copies/ml	1	0	No		Regular partner (age unknown) confirmed positive and initiated on ART **Couple separated**
**8**	Female	27	Chronic	4.22	Not detected	1	0	Yes	0/1	Regular partner (age unknown) confirmed negative, not in an ongoing relationship
**9**	Female	28	Chronic	6.42	<1.60copies/ml	1	0	No		Spouse (age unknown) reported to be known positive and on treatment
**10**	Female	32	Chronic	4.23	Not detected	1	0	Passive referral	1/1	Regular partner (26 years) newly diagnosed with chronic HIV. **Couple separated**
**11**	Female	39	Chronic	5.05	<1.60copies/ml	1	0	Yes		Spouse (age unknown) self-reported negative
**1**	Male	20	Chronic	4.85	<1.60copies/ml	1	0	No		Status unknown
**2**	Male	24	Chronic	4.50	4.47	2	2	Yes		Spouse (age unknown) self-reported negative; regular & casual partners’ status unknown **Couple separated**
**3**	Male	24	Chronic	4.76	Not detected	1	0	Yes		Spouse (age unknown) self-reported positive and on ART
**4**	Male	30	Chronic	4.90	Not detected	1	0	No		Regular partner (age unknown) self-reported positive and on ART
**5**	Male	36	Chronic	3.20	Not detected	2	2	Yes		Spouse (age unknown) self-reported negative; “other” regular & casual partners’ status unknown
**6**	Male	39	Chronic	5.47	<1.60copies/ml	0	1	No		Casual partner (age unknown) status unknown
**7**	Male	39	Chronic	4.14	<1.60copies/ml	2	0	Yes	1/1	First wife (age unknown) status unknown. Regular partner (42 years) on ART **Couple separated** (partner of male #7)
**Partners enrolled at research clinic and initiated on ART**										
**1**	Male	26	Chronic	5.01	<1.60copies/ml	1	1	NA	NA	Casual partner status unknown. **Couple separated** (partner of female #10)
**2**	Female	42	Chronic	3.84	<1.60copies/ml	1	0	NA	NA	**Couple separated** (partner of female #7)

Abbreviations: HPN = HIV partner notification, LTFU = lost to follow-up, ND = not detected.

^1^ Elite controller.

### PNS outcomes

Table 2 also shows the outcomes of PNS among the 18 index patients (12 of 18 consented for PNS). Overall, 28 partners (22 regular and 6 casual) were reported among 17 index patients (one index patient reported not to be sexually active), of whom 7 regular partners (two women and five men) agreed to be tested at the research clinic. One male and one female partner were newly diagnosed and started ART immediately. Both achieved viral suppression by month 6. Five partners (four men and one woman) tested HIV negative. All men who tested negative started PrEP on the day of testing, among them one partner of an AHI index patient; the woman who tested negative declined PrEP. Three participants completed PrEP at month 6 as recommended per Kenyan guidelines, while one participant discontinued PrEP at month 4.

### Partnership dissolution over time

Over the 9-month observation period, 5 of 18 index participants, including 2 of 7 index participants whose partner was tested, reported that their partnership had dissolved. These included one of the two couples with AHI, one discordant couple with the female partner not on PrEP, and three concordant HIV positive couples who were in follow up at the research clinic. (Table 2).

### Emergent themes

In qualitative analysis, we identified the following key emerging themes centered around time, patients’ situations and coping mechanisms during the 9 months following their HIV diagnosis. Quotes illustrating patients’ experiences are included in the description below.

#### Factors influencing status acceptance

*Acceptance of and commitment to ART*. Patients’ primary symptoms such as fever, headache, loss of appetite, and fatigue were initially thought to be associated with malaria or flu, which prompted their clinic attendance and request for medications to treat these symptoms. If patients had not been tested for HIV by chance, they could have gone much longer without being diagnosed, resulting in delayed ART initiation and posing a risk for transmission to uninfected partners. However, across interviews, many participants described how their initial distress at learning about their HIV status was alleviated through initiating ART. We quote a 24-year old female who did not seem a likely candidate for HIV testing because she did not consider herself at risk, but who immediately felt reassured when her symptoms were explained and treated:

*“I thought I was suffering from malaria and pneumonia*… *I was feeling tired*, *my stomach was aching*, *and I was just feeling pain all over*. *When I went to the chief’s clinic*, *they [healthcare providers] compared some of the symptoms that I had*… *I was told that I was going to be (HIV) tested*. *I got to know my HIV status and I got HIV treatment*.*”*– 24-year-old female, 1^st^ interview

Another participant reflected on his strong emotional reaction after receiving an HIV diagnosis, including depression and thoughts of ending it all. He recalled that ART initiation treated not just his symptoms but conferred greater peace of mind, allowing him to accept his status and move on with life:

*“Generally*, *the [ART] medication has had a positive effect on me*, *especially health wise*. *It also has made my mind relax in some way*. *Because when I was tested and was told I have the virus if it were not for using the drugs*, *I would have killed myself because since I was in school*, *I had said at the moment I will have the virus … once I know I have the virus*, *that will be my last day*, *I will kill myself*. *In whichever way*, *I would have killed myself*. *But the way I got information there I said it is okay let me continue*.*”*– 24-year-old male, recently diagnosed prevalent case, 3^rd^ interview

Similarly, partners of newly diagnosed index patients found that initiating PrEP medications provided a sense of comfort with their partners’ diagnosis and with the reality of being in a serodiscordant relationship. A partner of one of the AHI cases felt that PrEP as a strategy to prevent transmission, *together with his confidence in recommendations from the healthcare provider*, *motivated PrEP adherence during* his partner’s period of increased infectiousness due to her high viral load:

*“It [PrEP] is very good*. *It assisted me so much*. *That is why am still negative*… *I could have been infected too because of this [wife’s] high viral load*… *I took [PrEP] on a daily basis and I didn’t miss even for a single day*. *Daktari kuja kukuambia ni sawa [it is okay if a doctor comes and tells you]*.*”–* 36-year-old male on PrEP, 4^th^ interview

*Attaining viral suppression*. In the four discordant couples, PrEP use seemed an acceptable approach to reduce transmission risk until the infected partner attained viral *suppression*. In general, the newly diagnosed index patients whose partners were not known to be HIV infected were well aware of the fact that higher viral load increased transmission risk within serodiscordant couples. The following quote was from a participant whose partner declined PrEP, but who had confidence in his ability to use condoms until he achieved viral suppression:

*“When I was tested*, *my viral load was high*. *Both of us [my wife and I] decided that I should take the drugs [ART] and use protection [condoms] to prevent transmission and a pregnancy*… *You know*, *facts will always remain facts so is the fact that I am HIV positive*, *then I think you should stick to the medication*. *Starting medication immediately at least gives you time to change*… *to make up*, *what do we call this*…? *It helps with the acceptance [of HIV status]*.*”* 36-year-old male, 1^st^ interview

Sixteen index patients were able to start and continue with ART, which was later evidenced in patients’ viral load decreases at month 6. Participants in HIV-positive seroconcordant relationships realized that they would have had a poorer prognosis if untreated. They apprehended that HIV is long-term and life-altering. Optimistic developments were carefully expressed in different ways over time. The following quote offers a perspective on how viral load results and provider support helped the participant cope with HIV with more resilience and ability to maintain hope:

*“Personally*, *I think I am moving on well*, *I am taking my drugs [ART] as instructed and I can see my life is improving*. *There is a change*. *One of the advantages is that it [ART] reduces the viral load and it helps you to live longer… Since I got tested*, *I was able to open up my heart*, *I went to the facility with her [index] and after the viral load result was out*, *we were given guidance and counselling on how important it is not to be able to detect [the] viral load*, *and we followed whatever we were told [by the healthcare provider]*.*–* 30-year-old male, 4^th^ interview

The fact that viral load monitoring was readily available in the clinic site may have increased confidence in treatment. Baseline viral load measures helped patients understand that they had the virus and were infectious, and simultaneously increased patients’ understanding of the “undetectable equals untransmittable” concept:

*“Because I was very weak*, *I couldn’t take them [ART]*, *but daktari [clinician] told me that even when I am sick*, *I should take ART*, *because the drugs stop the virus from multiplying*. *Now my viral load is not detectable anymore*.*”*– 23-year-old female, 4^th^ interview

*Recognition of partner supportive role*. Participants who accepted partner notification services for HIV reported disclosing to their primary sexual partner on the same day of diagnosis. For most participants, disclosure came with a sense of relief and hope for support, but it also had many challenges, especially in the context of discovering that they were serodiscordant in their relationship. The following quote shows how a newly diagnosed woman and her husband overcame initial fear, anger and intense negative reactions:

*“I showed him [husband] the medication [ART]*, *and his negative feelings really shocked me*, *he hadn’t expect to receive such information*…, *that night*, *out of anger*, *he told me to go back to the people who infected me*…*”*– 24-year-old female, 2^nd^ interview

Instead of letting the reaction described above overwhelm her and undermine the relationship, the participant embraced patience and encouraged her partner to seek HIV testing following her own diagnosis. Her partner’s negative HIV result and subsequent willingness to take PrEP re-established trust and commitment:

*“So*, *after he [husband] tested negative*, *he didn’t want to end our relationship because he knew me*, *and he trusted me*…*He was really hurt*, *he couldn’t believe what I was telling him*, *but he just let it go*. *He has been very supportive ever since*, *and even reminds me to take my medication*.*”*–the same 24-year-old female, 3^rd^ interview

Although the advantage of the TMP study was that individuals interacted with healthcare providers on a regular basis, the changing dynamics among couples were unpredictable. Despite working through the initial tension following her diagnosis, the same participant quoted above mentioned in her 9-month interview that she and her husband had separated.

*Focus on faith*, *meaning and support*. Acknowledging the existence of loss, pain and suffering, participants turned to religion for personal meaning and comfort. While the challenges following the HIV diagnosis left some participants feeling guilt and shame, others overcame these challenges through their faith. Such participants believed that ultimately God decides everything and could remove their psychological burden. One man who relied on faith explained:

*“I left home to come to the hospital*, *and I prayed to God that I will accept whatever outcome*. *After the outcome*, *I accepted and was satisfied*, *it cleansed my soul*… .*”*– 30-year-old male, 1^st^ interview

Individuals who turned to faith after diagnosis hoped that God would increase the effectiveness of ART and reward them. One participant combined his belief in God with trust in biomedicine.

*“Yeah*, *I am taking my medication because*, *when I learned about my HIV status*, *it pushed me closer to God*. *I have a lot of hope in God more than in myself*…*I will stick to the drugs and also stick to my prayers*… *I believe that there is nothing that is impossible with God*.*”* 39-year-old male, 3^rd^ interview

For all participants, the journey to acceptance of HIV was not easy, but identification of an encouraging confidant helped with status acceptance. Creation of a support system, or a sense of being part of a faith community was central to patients’ well-being. A male participant described how after his HIV diagnosis, he initially thought of death and felt defeated. Actively seeking advice from a number of sources of support helped them broaden their perspective and see the light at the end of the tunnel.

*“During the beginning of the process*, *when I was a bit depressed*, *I was constantly looking for advice from everywhere*, *my friends*, *my family*, *sometimes even on YouTube channels and social gatherings*. *I think it [seeking advice] has been helpful to open up my eyes to the world at large*.*”–* 20-year-old male, 3^rd^ interview

Other participants noted how connection with other PLWH bestowed a sense of solidarity with others and gave them hope and renewed commitment to life:

*“I remember being very sad at the beginning*. *After knowing that I am not alone and there are other people who are suffering from the same disease even within my community*, *that gave me a sign of relief*. *And since discovering that*, *I decided to take my medication and continue to live my life*.*”*–, 23-year-old female, week 2

**Fig 3:**
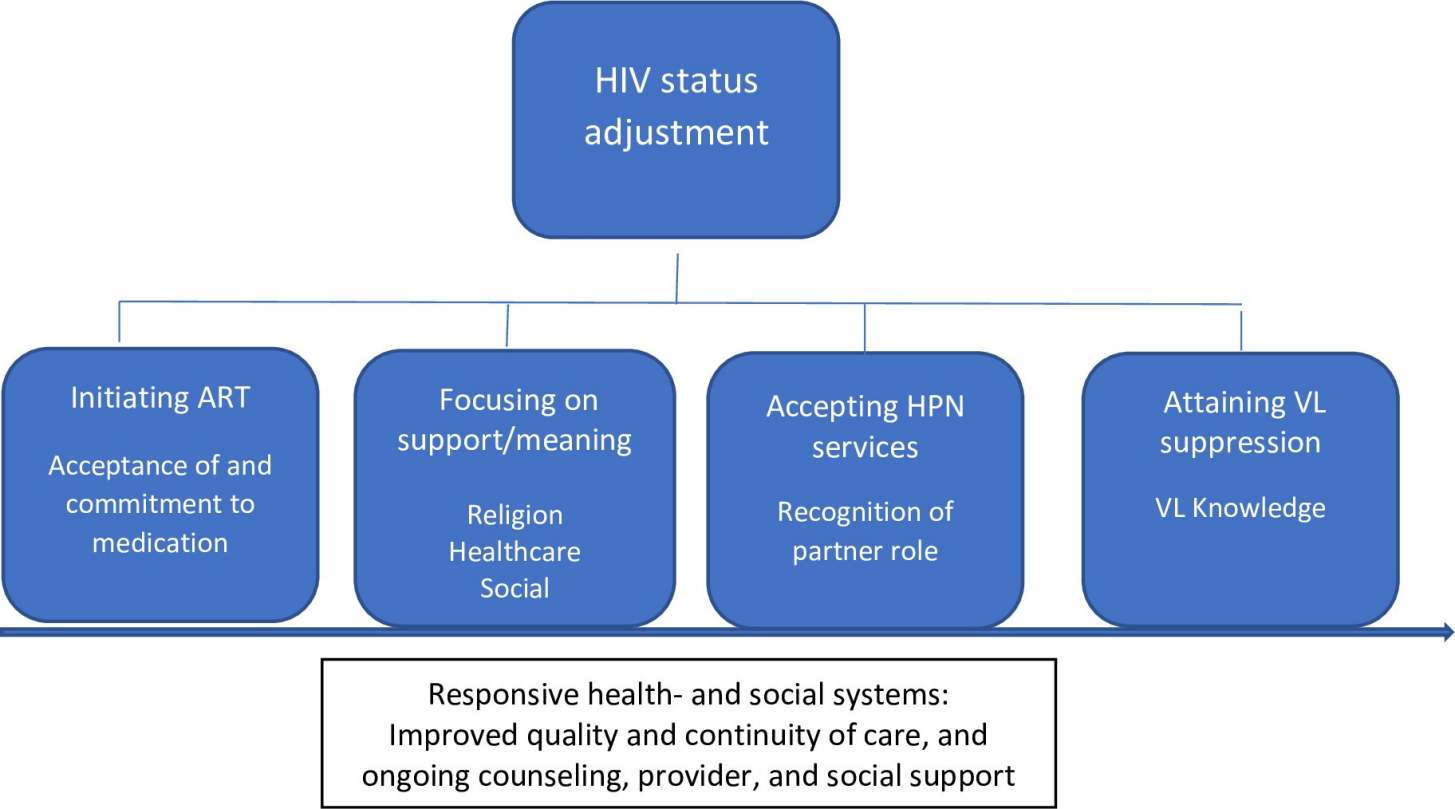
Conceptual model to facilitate HIV status acceptance by newly diagnosed patients with acute or chronic HIV infection and their primary partners.

Fig 3 summarizes the findings in an adjusted preliminary conceptual framework to further facilitate HIV adjustment for acute and newly diagnosed chronic patients. Although the core concepts of this framework may operate sequentially for many patients, these concepts may not necessarily follow a systematic temporal or linear trajectory for all patients.

## Discussion

We gathered in-depth narratives from young Kenyan adults who sought care for unexplained health symptoms and, in the process, were unexpectedly diagnosed with HIV, with our analytic focus on their narratives concerning how they had to come to terms with their HIV status over time. Experiences recalled by index participants and their partners provided insights into the specific challenges, participants faced after learning of their diagnosis and how they addressed (or did not address) these challenges over time. This research provides additional evidence for the need to develop client-centered interventions in Kenya to support individuals who receive a new HIV diagnosis, in order to support the adjustment process for managing their own health and maintaining healthy relationship with primary partners.

Findings from this analysis correspond with key elements of a previous study that proposed a conceptual framework for supporting the needs of Kenyan patients diagnosed in the acute or early phase of HIV [13]. The framework (corresponding to the acronym U-ADAPT) emphasized the critical time period after receiving a diagnosis of acute or early HIV, in order to help the patient *understand* and *accept* their diagnosis, *develop* healthy coping strategies, *adjust* to the reality of being a person living with HIV, and *protect* oneself and others through *treatment*. In keeping with this model, both the patients diagnosed with acute or with chronic HIV infection in our sample highlighted the importance of acceptance of and commitment to medication, attaining viral suppression, and recognizing the importance of their partners’ role post-diagnosis, i.e. disclosing and seeking support from their partner after diagnosis. With respect to coping strategies, many participants emphasized the importance of finding meaning and support through faith and community networks. Discussing experiences with other PLWH was also important in helping participants adjust to their new diagnosis and accept their need for lifelong HIV treatment. Findings from this research also correspond with other frameworks and research models on resilience regarding people’s ability to cope with recent HIV infection and persist through challenging circumstances [17]. Participants in our sample characterized the time after HIV diagnosis as a dynamic period in which they dealt with confusion, contradictions, and ruptures in their lives and relationships, but found relief and comfort through support from others, guidance from their providers, and encouragement in their faith.

This study describes participants’ trajectory from initial diagnosis to the time point in which they achieved viral suppression was marked by themes related to ART initiation and HIV status acceptance, disclosure to and engagement with partners to engender support, seeking out community support, and finding meaning through faith. In particular, narrative findings underscored the importance of connection with primary partners and supportive individuals who can empathize with the burden and reality of living with HIV. HIV status disclosure to one’s partner–either on one’s own or through PNS procedures–was a challenging but important step in this process [14]. Some of index participants’ partners, initiated PrEP as a means to protect themselves, and PrEP was part of the process of status acceptance for these couples. Additionally, the availability of community support networks provided an environment that encouraged and enabled adherence.

Our interviews with this sample, consisting of 18–39 years old individuals who had sought care for symptoms, suggested that newly diagnosed PLWH in Kenya, after having been initially in shock, can develop more openness and acceptance with their HIV status over time, and that this process is optimized by having a supportive personal and social environment as well as access to clinical care. This aligns with previous reports from Kenya [18], especially when it comes to younger people, and with other studies in Sub-Saharan Africa indicating that additional counseling can prepare patients and their partners to better cope and adjust with HIV status acceptance. In the context of PNS, tailored and individualized strategies combined with healthcare support increased the chance of partner disclosure. Twelve of 18 index participants in our sample agreed to PNS, while over one in four of the partnerships dissolved in the course of nine months. This indicates an urgent need for approaches to support both newly diagnosed patients and their primary partners, emphasizing the potential benefits of mutual status disclosure, and HIV prevention or care appropriate to the status of each individual.

For many people in SSA, the belief that HIV is an incurable, fatal, and a blameworthy disease has contributed to the need for personal secrecy about one’s HIV status [19]. Yet, disclosure is an important part of HIV status transition and acceptance process. Simultaneously, individual, community, and societal norms impact these processes differently across different population groups based on levels of stigma and access to support resources. Through the participants’ narratives we learned that religion was often a main source of support, assisting with the psychological toll of diagnosis and acceptance of medication. In conjunction with religious belief was recognition of a support system and a sense of community. The feeling of being part of a group generated a more positive attitude of participants themselves and their relationship with others over time.

The findings demonstrate the need for HIV care guidelines to focus on mental health among newly diagnosed people. While immediately offering ARTs to individuals’ post-diagnosis is important to achieve viral suppression, there also needs to be a focus on systems to enhance support/counseling interventions for newly diagnosed PLWH in this region, including an emphasis on patients’ primary relationships.

As the interviews were anchored in real-life situations, limitations to this research must be acknowledged. First, the sample size was relatively small and not inclusive of all newly diagnosed patients in the study. Second, the qualitative nature of the data limits the ability to generalize across similar populations, though we believe that the validity of content is high since the use of follow-up interviews allowed for revisiting patients’ experiences. Third, qualitative interviews were conducted in the context of participation in a longitudinal cohort study, in which access to trained medical providers and counselors, medications, and viral load monitoring was ensured, which unfortunately does not reflect general availability and quality of services for PLWH in this region.

## Conclusion

To support HIV newly diagnosed patients, health and social systems must be more responsive to the needs of both individuals and couples (comprising index patients and their primary partners), while improving provider training and strengthening continuity of care across facilities. Promising interventions need to be co-designed with health providers, counselors, community and religious representatives, and carefully tested for unintended negative consequences and assuring the potential for sustainable scale-up. The following recommendations can possibly serve as potentially effective interventions to facilitate healthy adjustment to a new HIV diagnosis, whether acute or chronic: 1) improve patients’ understanding of the benefits of early HIV diagnosis and ART; 2) conduct multiple counseling sessions, preferably together with the primary partner, to enable safer sex choices and use of PrEP if indicated, and most importantly to allow for facilitated discussions within couples; 3) understand the contextual environment or the spaces where disclosure occurs and equip patients with skills to disclose in a safe manner; and 4) encourage patients to gain various forms of support such as support for treatment adherence, emotional support for greater well-being, and messaging on U = U and how treatment protects others from HIV.
